# Audio-Tactile Rendering: A Review on Technology and Methods to Convey Musical Information through the Sense of Touch

**DOI:** 10.3390/s21196575

**Published:** 2021-09-30

**Authors:** Byron Remache-Vinueza, Andrés Trujillo-León, Mireya Zapata, Fabián Sarmiento-Ortiz, Fernando Vidal-Verdú

**Affiliations:** 1 Departamento de Electrónica, Universidad de Málaga, 29071 Málaga, Spain; atrujilloleon@uma.es (A.T.-L.); fvidal@uma.es (F.V.-V.); 2 SISAu Research Group, Facultad de Ingeniería y Tecnologías de la Información y la Comunicación, Universidad Tecnológica Indoamérica, Quito 170103, Ecuador; fabiansarmiento@uti.edu.ec; 3 Instituto de Investigación Biomédica de Málaga (IBIMA), 29010 Málaga, Spain; 4 Research Center of Mechatronics and Interactive Systems—MIST, Facultad de Ingeniería y Tecnologías de la Información y la Comunicación, Universidad Tecnológica Indoamérica, Quito 170103, Ecuador; mireyazapata@uti.edu.ec

**Keywords:** human computer interaction, haptic music player, musical haptics, musical haptic wearables, sensory substitution systems, tactile rendering, vibrotactile feedback, vibrotactile music composition

## Abstract

Tactile rendering has been implemented in digital musical instruments (DMIs) to offer the musician haptic feedback that enhances his/her music playing experience. Recently, this implementation has expanded to the development of sensory substitution systems known as haptic music players (HMPs) to give the opportunity of experiencing music through touch to the hearing impaired. These devices may also be conceived as vibrotactile music players to enrich music listening activities. In this review, technology and methods to render musical information by means of vibrotactile stimuli are systematically studied. The methodology used to find out relevant literature is first outlined, and a preliminary classification of musical haptics is proposed. A comparison between different technologies and methods for vibrotactile rendering is performed to later organize the information according to the type of HMP. Limitations and advantages are highlighted to find out opportunities for future research. Likewise, methods for music audio-tactile rendering (ATR) are analyzed and, finally, strategies to compose for the sense of touch are summarized. This review is intended for researchers in the fields of haptics, assistive technologies, music, psychology, and human–computer interaction as well as artists that may make use of it as a reference to develop upcoming research on HMPs and ATR.

## 1. Introduction

Human beings perceive the world and interact with it through the senses. The sense of touch, for instance, enables humans to perceive temperature and roughness of a surface. According to the authors of [1], tactile stimuli are perceived through a series of complex mechanisms which are part of the somatosensory system. More specifically, mechanoreceptors allow us to feel tactile stimulation in the skin, whereas proprioceptors in joints, muscles, and ligaments enable kinesthesia (the ability to feel weight, location, and position of limbs) [2]. There are four channels associated with mechanoreception: Pacinian (P), Non-Pacinian I (NP-I), Non-Pacinian II (NP-II), and Non-Pacinian III (NP-III) which activate depending on the frequency of the stimuli and according to what is shown in Table 1. Moreover, it is well known that fast afferent I (FAF-I) and fast afferent II (FAFII) physiological types, into the P and NP-I channels, respectively, are responsible for processing vibrotactile stimuli [3]. The limits for vibrotactile stimuli perception have been investigated and established from values as low as 0.3 Hz to a maximum of 1000 Hz [4,5,6]. Best perceived frequencies varies depending on the part of the body and the intensity of the vibrations (i.e., amplitude). In [7], the researchers compared thresholds for the fingertip, forearm, and abdomen, and found out that the fingertip is more sensitive as it allows best perception of stimuli around the same frequency but at lower intensity; less energy is required for vibrotactile stimuli to be perceived in the fingertip. Furthermore, an overall peak frequency of 250 Hz has been suggested by the authors of [8]; however, as sensitivity varies from one part of the body to another, it would probably be better to establish an overall best sensitivity range such as the one proposed by [5], ranging from 50 Hz to 500 Hz, or depending on the part of the body such as the range for the head suggested by [9] which lays between 32 Hz and 64 Hz.

Another parameter that defines vibrotactile perception is spatial resolution, which refers to the ability to recognize stimuli presented in different parts of the body. According to Goldstein [10], spatial resolution also varies depending on the location of the stimuli and ranges, on average, from 10 mm in the hand and lips, to over 40 mm in the back and calf. Moreover, location recognition of two vibratory stimuli depends on two temporal factors: duration of stimuli and inter-stimulus onset asynchrony (ISOA) (i.e., when each actuator is activated and deactivated), and acuity decreases as number of stimuli presented increases [3]. Thus, it is clear that frequency, amplitude, location in the body, number of stimuli, and ISOA are important parameters to take into account when determining vibrotactile thresholds, and should be explored in accordance to the application or experimental setup. To find out more about tactile perception thresholds check the works in [7,10,11,12]. Other relevant parameters that affect vibrotactile perception are frequency change discrimination (pitch change) and signal rendering, which will be later discussed in the context of audio-tactile rendering (ATR).

### 1.1. Vibrotactile Feedback

Touch has been successfully exploited as an alternative channel to communicate information by means of haptic interfaces. Human–computer haptic interfaces (HCHIs) are devices that offer the user force feedback to enable interaction with technology [13]. This force may be generated by vibrating actuators in contact with the skin. Probably, the most common implementation is vibratory stimuli in cellphones to notify users regarding incoming messages, incoming calls, and alarms. HCHIs are also used as sensory substitution systems (SSSs) to convey information to the hearing impaired or blind individuals [3]. For modality translation to be successful, it is necessary to follow a systematic process that begins with the identification of key information features from the parent modality, then the information processing or mapping is performed, and finally information is presented adequately in the alternative modality [14,15]. Feedback may be available to configure parameters of vibrotactile stimuli based on user perception. When the haptic interface conveys tactile information through vibrations it may also be called vibrotactile interface [16], vibrotactile display [17], or vibrotactile sensory substitution system [18], and the information flow between it and the user is usually known as vibrotactile feedback. Some references to review the use of the concept of vibrotactile feedback are found in [2,3,8,9,19].

### 1.2. Musical Haptics

The term musical haptics was first introduced by Papetti in 2018 [20] and refers to the use of force feedback to enhance the digital musical instrument (DMI) playing or the music listening experience. As proposed in [21], haptic technology is suitable to communicate musical information and enrich the experience of listening to music, live or recorded. For the case of music listening, vibrotactile feedback is the usual selected mechanism to convey musical information through the skin. Although in [5] the author observed that not every aspect of music may be mapped from audio to vibrations, various researchers (see, e.g., in [2,22,23]) have demonstrated that strategies may be outlined to extend the possibilities to render music characteristics to vibratory stimuli. In addition, the Haptic and Audio Interaction Design Workshop (HAID), discontinued since 2013, was reactivated in 2019 due to the increased necessity of a venue to connect the research fields of haptics, audio, and human–computer interaction (HCI) [24]. According to the authors of [20], there are research opportunities in musical haptics to enhance vibrotactile feedback, specially on sensors, actuators, portability, energy efficiency and signal processing. On the other hand, by the time of this review there is no established methodology to evaluate the user experience, so future consensus would be necessary to make musical haptic technology scalable.

### 1.3. Structure of the Review

In Section 2, a previous publication with review content about musical haptics is shortly described and then the tools and strategies for searching are outlined. In Section 3, types of haptic music players (HMPs) are described and systematized. In Section 4, methods for tactile rendering of music are studied. In Section 5, strategies to compose for the sense of touch are contrasted. Finally, in Section 6, a discussion regarding current challenges and future work is added, and the most important conclusions are drawn.

## 2. Methodology

### 2.1. Previous Reviews

**Figure 1:**
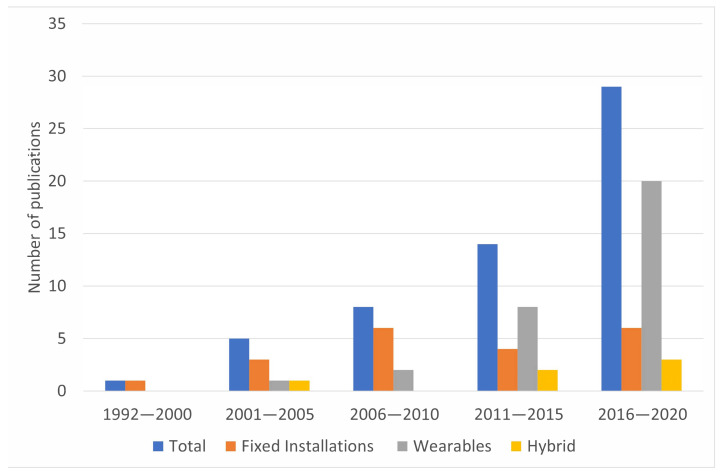
Number of HMP publications since 1992 until 2020.

The book “Musical Haptics” [20], published in 2018, compiles works on haptic technology applied to DMIs and one focused on a HMP named “Auditory-Tactile Experience of Music” by Merchel and Altinsoy [25]. This work focuses on the evaluation of music listening experience with vibrotactile feedback using a haptic seat. Other related works are referenced in [14,15,18,26,27,28,29,30,31,32,33,34]; but no systematic exploration was observed. In addition, it was verified that about 51% of the HMP literature was published in the last 5 years (Figure 1), and the literature covered in [20] represents just ~23% of the total literature published at this time.

### 2.2. Review Method

**Figure 2:**
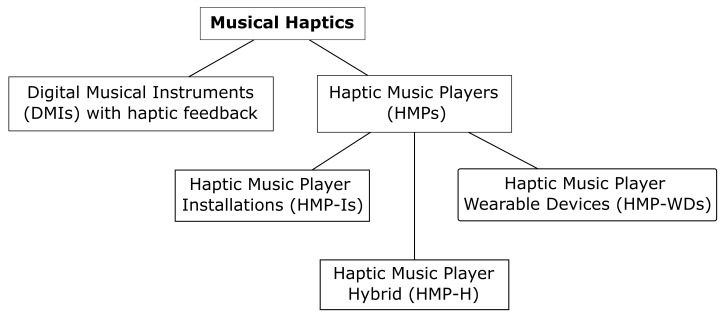
Preliminary classification of musical haptics.

To narrow the search process, musical haptics was divided into two groups: haptic displays to enhance music playing of DMIs and haptic displays to play haptic music (i.e., HMPs), as shown in Figure 2. This review focuses on the right branch of the proposed classification corresponding to HMPs. Due to the trans-disciplinary nature of the topic, databases Scopus, Web of Science and PubMed as well as search engine Google Scholar, were used to look for literature. The keywords used to search are: haptics, music, vibrotactile, player, skin, composition, haptic chair, haptic wearable, sensory substitution, and the following combinations:

- Musical haptics;
- Haptic music;
- Haptic music player;
- Musical haptic wearables;
- Vibrotactile music;
- Vibrotactile composition;
- Vibrotactile music composition;
- Vibrotactile music player;
- Skin music;
- Skin music player;
- Music sensory substitution system.

To extract information of literature Mendeley web importer for Google Chrome was used. Metadata were managed in the library of Mendeley (i.e., web and desktop) and synced with Overleaf for referencing in the manuscript. This workflow allowed reliable and efficient reference management.

## 3. Haptic Music Player (HMP)

### 3.1. Architecture

To provide vibrotactile rendering of musical elements four main components are necessary: audio signal processing software, digital to analog converter (DAC), amplifier(s), and actuator(s). The information flow starts with an audio signal which may be extracted from the local storage or from an audio transducer (i.e., a microphone). The audio signal is then processed to extract musical information and then is translated to vibratory signals. The intensity of signals is controlled using an amplifier and the resulting analog signals are finally sent to the vibrating actuators that will be in contact with the user’s skin. The interface may provide interaction options such as knobs or buttons so that the user can have certain level of control over the vibrating stimuli [26,35]. Depending on the goals of the research the installation may have sensory modality variations: haptic feedback, haptic-auditory feedback, haptic-visual feedback, or haptic-auditory-visual feedback. After reviewing the literature, it was found that actuators and signal processing are the main focus of research. Actuators will be studied according to the technologies and the way they are attached to the skin. Signal processing will be analyzed in Section 4 and Section 5, according to the techniques used to translate music features to vibrotactile stimuli and as a strategy to compose for the sense of touch.

### 3.2. Actuators

Actuators are vibrating devices that are in contact with the user to convey information by means of vibrotactile stimuli. The types of actuators usually encountered in musical vibrotactile displays are listed in the following.

#### 3.2.1. Voice Coil Actuator (VCA)

VCAs are the most commonly used actuators for conveying musical information through vibrotactile stimuli, as the audio signal may be used directly to activate them with little or no additional signal processing; pitch translates to frequency and loudness translates to intensity of vibrations [8]. VCAs are efficient low-cost devices that have good response to amplitude changes of signals [2].

#### 3.2.2. Linear Resonant Actuator (LRA)

LRAs are suitable for vibrotactile applications as they work in similar way as loudspeakers and VCAs [36]. They have a smaller frequency response bandwidth [37], thus suitability for a given application must be evaluated taking into account tactile perception thresholds.

#### 3.2.3. Eccentric Rotating Mass (ERM)

ERMs are rotating motors with an eccentric mass attached to the shaft. These type of actuators are small and lightweight, which make them suitable to enhance portability of the haptic interface [18]. However, independent control of amplitude and frequency of vibrations is not possible. Although the investigators in [18] consider response of ERMs enough to convey information even through clothes, Hwang, Lee, and Choi [37] state that response to changes in dynamics of ERMs is slow. Thus, mapping of signals carrying temporal components of music such as rhythm to ERMs has to be performed carefully, as may not be accurate [8].

#### 3.2.4. Piezoelectric Actuator

Piezoelectric actuators, or piezo-buzzers, vibrate as a result of alternating displacements generated by changes in electric field. Even though they require specific electronics to be driven [20], piezoelectric actuators are flexible, low-cost, energy efficient, and have a wide operating frequency band.

#### 3.2.5. Dual Mode Actuator (DMA)

DMAs are small actuators that provide vibrotactile stimuli composed of two fundamental frequencies. The authors of [37] demonstrated that it is possible to render complex signals that users are able to recognize and evaluate as better compared with LRAs. In addition, computation for activation of DMA is easier and more efficient.

#### 3.2.6. Loudspeakers

Although loudspeakers are not thought as actuators, they generate vibrations that can convey musical information through the skin as demonstrated in [33,38,39,40,41]. Frequency response of loudspeakers by far overcome upper tactile perception threshold (e.g., up to 20 kHz), so subwoofers are usually selected for haptic applications. Working principle is similar to that of VCAs but the vibrations are obtained in a diaphragm. Pitch and loudness are also translated to frequency and intensity, respectively [8]. Loudspeakers also allow independent control of frequency and intensity [3], which is desirable to convey signals that carry more complex musical information such as timbre or melody.

Check Section 3.3 for references on types of actuators implemented by type of actuator attachment mechanism.

#### 3.2.7. Actuator Selection

According to Giordano and Wanderley [3], there are four main criteria to select actuators for musical applications: the role of the vibrotactile stimuli in the interface, size, energy consumption, and the kind of information that will be conveyed. For the case of HMPs, vibrations are intended to stimulate the skin according to a musical composition to convey music to the hearing impaired, to enhance the music listening experience, or to convey vibrotactile music compositions (VMCs). Regarding size and energy consumption, bigger actuators such as speakers and some voice coil actuators may need amplification which results in higher energy consumption. In addition, according to Petry, Huber, and Nanayakkara [8], resolution of stimuli perception may increase as the area covered by the actuator increases. However, as explained in [15], the increment in area in contact with the skin at some point will result on diffusion of stimuli, thus recognition of location of vibrotactile source may be more difficult [9]. Therefore, a balance between size and stimuli location will allow high resolution tactile rendering. Furthermore, enhancing performance of actuators at low frequencies is desirable as the frequency range for best perceptible vibrations is lower than that of audio. Innovations such as the string-motor actuator proposed in [42] allows to cover a wider area efficiently but effectiveness to render specific music features, such as melody or timbre, remains unclear. On the other hand, the DMA proposed in [37] allows complex signal mapping covering a smaller area, which suggests that balance between size and stimuli resolution is achievable. Due to the difference on sensitivity of human skin along the body, choice must also be based on a careful analysis of frequency perception thresholds and responsiveness of the actuator. Although no specific strategy to select actuators for HMPs has been encountered by the time of this review, design considerations found in [43,44] are useful for preliminary design. Selection of actuators represents an opportunity for future research, specially for comparison between types of actuators, advantages, and limitations regarding vibrotactile rendering of musical features. In summary, best actuator selection combines low-cost and lightweight design, enhanced performance at low frequencies and a good balance between size and vibrotactile stimulation.

### 3.3. Actuator Attachment Mechanism

To communicate vibrating stimuli, actuators are attached to the user in different ways and in different parts of the body. Contact mechanisms may be organized into three groups: haptic installations, haptic wearables, and hybrid.

#### 3.3.1. Haptic Music Player-Installation (HMP-I)

**Figure 3:**
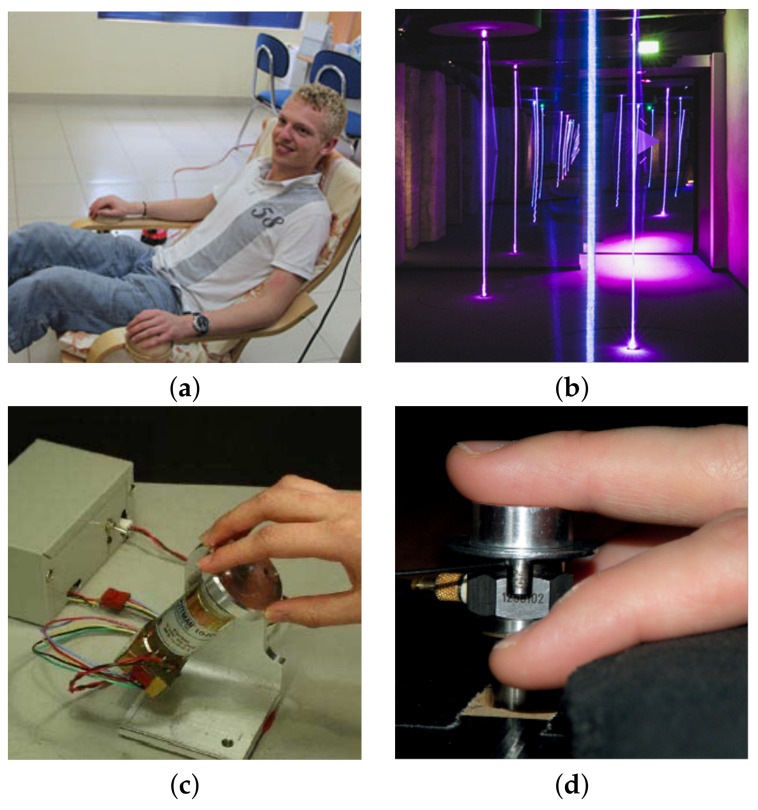
Examples of haptic player installations (HMPIs): (**a**) Deaf user experiencing music in a haptic chair, retrieved from in [8]. (**b**) Multimodal platform, retrieved from in [49]. (**c**) Desktop haptic knob installation, retrieved from in [50]. (**d**) Desktop haptic button installation, retrieved from in [51].

HMP-Is are fixed setups for analyzing psychophysical responses of hearing-impaired users to musical vibrotactile stimuli, although there are experimental setups designed to study responses of hearing individuals also. Haptic chairs are the most common installations found in the literature. The concept of using a chair as an HMP was patented by Komatzu in 2002 [45] and has been explored since then by various authors, as shown in Table 2. The primary advantage of using a chair as a medium of vibrotactile communication is the extended area available to spatialize stimuli. Space can be used as a vibrotactile music composition resource to compensate the limitations of tactile perception [15]. While sitting, the user is provided with vibrotactile feedback underside of the seat, the back, the seat arms, or the feet, while other sensory channels may complement feedback such as auditory, visual or both, depending on the purpose of the investigation. Some limitations of haptic chairs are portability and customization, as the user may not be able to change the location of the chair or the location of the actuators with ease. Important factors to be considered in the design of a haptic chair are bone conduction and airborne conduction, as they may be augmented through the structure of the chair. Perception of vibrotactile stimuli can be affected by bone conduction [46,47], and should be considered mainly if psychophysical evaluations are involved. High-frequency and high-intensity vibrations may produce not only audible resonance, but also vibration of internal organs such as the viscera whose excitation has been associated with emotion [15]. Bone conduction may be treated as interference in some cases, for instance when the vibrotactile stimuli needs to be assessed isolated from auditory perception (i.e., audible stimuli resulting from bone conduction may reach the ear and affect the results) [23]; however, it may be also desirable, for instance, to enhance the music listening experience as proposed by Sakuragi in [46]. These parameters have been taken into account in design stages in [25,30,48], as the body related transfer function (BRTF) similar to the head related transfer function (HRTF) in auditory studies. Other types of installations are multimodal platforms for user experience evaluation [49] and fixed desktop devices such as knobs [50] and buttons [51] for vibrotactile music perception evaluation, as shown in Figure 3.

#### 3.3.2. Haptic Music Player-Wearable Device (HMP-WD)

**Figure 4:**
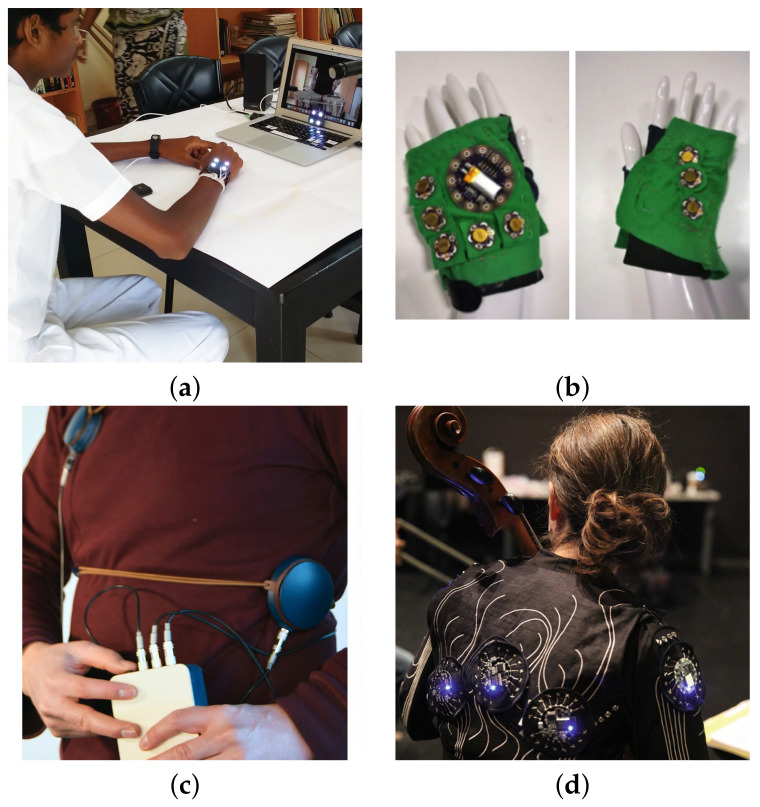
Examples of haptic music player-wearable devices (HMP-WDs): (**a**) Deaf user experiencing music with a haptic bracelet MuSS-Bits, retrieved from in [19]. (**b**) Haptic glove, retrieved from in [61]. (**c**) Haptic belt Hedonic Haptic Player, retrieved from in [35]. (**d**) Haptic jacket Body:Suit:Score, retrieved from in [65].

The notion of wearability of HMPs was considered by Gunther in 2001 in his proposed vibrotactile compositional tool “Skinscape” [27]. Although the idea was not implemented, it evolved to a wearable whole body musical haptic interface presented in 2003 by Gunther and O’Modhrain [15]. Nevertheless, in 2018, Turchet [58] introduced the concept of musical haptic wearables for audiences or MHWAs, where vibrotactile feedback was transmitted via haptic garments that members of an audience could wear. In this review, the concept of HMP-WDs is proposed to generalize its implementation not only for listening experiences but also as SSSs for hearing impaired individuals. Even though Turchet describes basic requirements of MHWAs, such as embedded intelligence and wireless connectivity, some prototypes needed desktop installations because of the research stage at which the projects were, such as Gunther’s “Cutaneous grooves” whole body haptic interface, while others are musical haptic wearables intended for experimentation purposes only. Consequently, most works described in this section are not necessarily portable at the moment of publication, but have clear opportunities to lately become HMP-WDs. Starting from prototypes that cover a small skin surface, there are bracelets (Figure 4a), designed to be worn on the wrist (see, e.g., in [19,21,59,60]); gloves and mobile device mockups (Figure 4b), designed to be worn or held on the hands (see, e.g., in [37,61,62,63,64]); belts (Figure 4c), designed to be worn surrounding the body from the chest to the abdomen (see, e.g., in [8,35,41,42]); and jackets (Figure 4d), designed to be worn on the upper body with actuators usually located on the back, the front and the superior limbs (e.g., [38,65]). Other variations are whole body suits [15], and headphone type displays [39], but instances are scarce. As well as for HMP-Is, design of HMP-WDs requires consideration of frequency perception thresholds according to the part of the body where the device will be attached. Auditory feedback may be provided with almost no effect on portability but if adding visual feedback the user would be constrained to a reduced space, such as virtual reality (VR) or augmented reality (AR) installations. For effective portability, special attention must be focused on energy consumption to extend at maximum battery lifetime. Strategies such as pulse-width modulation (PWM) for efficient signal rendering may be combined with lightweight efficient actuators such as DMAs; although resulting increased noise should be assessed [36]. Additionally, if it is expected that the user wears the HMP, the appearance and user interface capabilities of the device are relevant and should be considered, as they are in [35,42,65]. Finally, bone and airborne conduction should be considered for high frequency-energy vibrations or when HMP-WDs are attached near the head or internal organs, as may influence vibrotactile perception. Belts and jackets are of particular consideration as they cover larger areas. Alternatively, Yamazaki et al. [42] showed that larger areas of the body may be covered using strings attached to vibrating motors instead of using the motors themselves to convey the vibrotactile stimuli. This is an innovative implementation that results in better transmission of low frequency vibrations than that obtained with linear actuators. Nevertheless, additional psychophysical exploration would be necessary considering tactile rendering of more specific musical features and the BRTF. Table 3 shows a systematic overview of research on HMP-WDs.

#### 3.3.3. Haptic Music Player-Hybrid (HMP-H)

HMP-H are setups that combine actuator contact mechanisms from HMP-Is and HMP-WDs. The use of bracelets, sleeves, and belts extends the area covered by fixed installations. In [75], for instance, electronic music artist Martin Garrix performs in a hybrid installation that features platforms, vibrating objects, touchable speakers, and jackets in order to convey musical information to hearing impaired individuals. Participants reported the experience as positive and energetic. Although expanding the area of a haptic installation may be desirable, for research purposes it may evolve to a HMP-I or HMP-WD. For instance, the model human cochlea proposed in [28] and ref. [14] started as a wearable device but ended up as a haptic chair [54], while the hybrid installation of Gunther [27] later became a wearable haptic whole body suit. Hybrid installations may include vibrotactile music input devices (VMIDs) such as the Vibrochord, designed and tested in [76,77], or the mobile device mock-up investigated in [62]. A VMID allows a performer to play vibrotactile music while the user perceives the stimuli in real-time. This vibrotactile music device is different from a DMI as it is intended for vibrotactile music execution or composition, and neither auditory stimuli is generated nor acoustic musical instrument sound simulated. Table 4 shows a systematic overview of research on HMP-H.

According to the literature reviewed in Table 2, Table 3 and Table 4, HMPs have evolved consistently during the last three decades. Although first ideas focused on fixed installations such as haptic chairs, it is clear that the tendency has been towards wearable technologies; about 54% of the total of publications corresponds to HMP-WDs with about 64% of them published in the last 5 years.

## 4. Audio-Tactile Rendering

High-quality rendering of audio features to vibrotactile stimuli would generate robust HMPs to aid the hearing impaired to feel music through touch and will effectively enhance the music listening experience of hearing individuals. Although translation of music features to vibrotactile stimuli is neither a straightforward nor a constrained task, it is possible to outline relevant mapping considerations. These considerations may be grouped according to the musical feature to be translated, being the most explored: rhythm, pitch, melody, timbre, and loudness.

### 4.1. Tactile Rendering of Rhythm

Rhythm may be defined as an auditory or visual pattern that repeats on time [77]. This ubiquitous musical feature may be perceived by multiple sensory channels such as visual, auditory, and touch. Moreover, rhythm recognition enhances when visual feedback is offered to the user [18,60]. Translation from auditory rhythmic patterns to vibrotactile stimuli in HMPs has been studied by various researchers (check Table 2, Table 3 and Table 4). It has been suggested that the sense of touch is able to recognize rhythm with ease [3,61], and that rhythmic patterns in vibrotactile music contributes to the experience in a greater proportion compared to other musical features [32,78]. Depending on the kind of music, rhythmic patters may have more presence in a specific frequency band, so one way to enhance vibrotactile rhythmic information is using filters [32,37]. For instance, in jazz music the bass or drums usually mark the rhythmic baseline, therefore a low-pass (high-shelf) filter may be used to cut high frequencies and enhance bass tones that carry rhythm. However, use of filters may affect the quality of the final vibrotactile composition [63]. Perception of rhythm with vibrotactile stimuli may depend on the type and size of actuator used. In [34], for instance, the authors found that covering a greater area of the body with the actuator allows users to better feel rhythm and energy of music. Rhythm may also be conceived from scratch. In [35], the authors create vibrotactile rhythmic patterns without processing audio signals from music. In this case, signals are synthesized to create short high intensity pulses which are later sequenced to create vibrotactile compositions. Other ways to create rhythmic patterns is using software instruments such as those found in digital audio workstations (DAW), where highly rhythmic instruments can be selected to create independent tracks [47], enabling a kind of vibrotactile orchestration tool. However, control over the signals sent to the actuators may be limited. On the other hand, when considering a HMP for hearing impaired individuals, it will be important to determine how they perceive rhythm as may be different to what a hearing person perceives [19]. The authors of [40] suggest that hearing-impaired users are able to identify rhythmic patterns in vibrotactile music, but no sufficient psychophysical evidence is presented to demonstrate that perception of vibrotactile rhythm is similar in deaf and hearing users.

### 4.2. Tactile Rendering of Pitch

**Figure 5:**
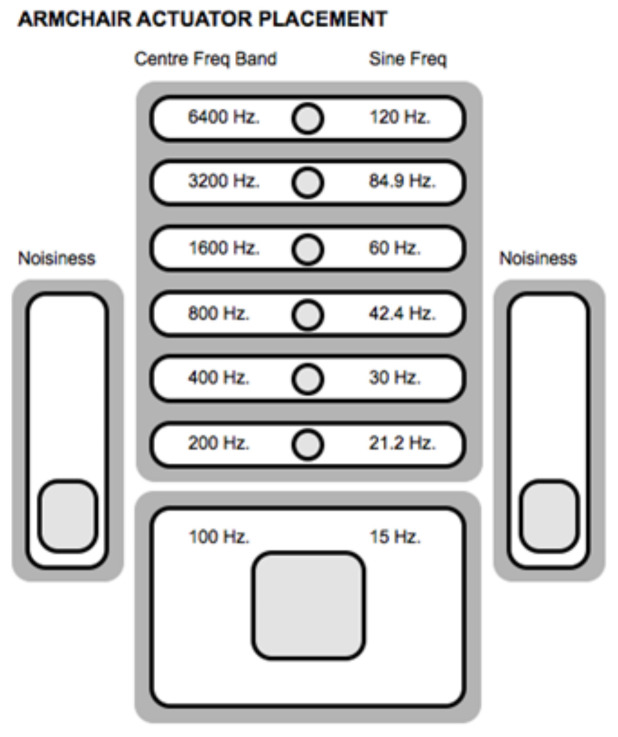
Spatialization of frequencies in a haptic chair, retrieved from in [32].

Rendering pitch to vibrotactile stimuli is a complex task as touch has frequency perception limitations that have already described. A simple way to translate pitch and loudness to vibrotactile stimuli is using speakers or VCAs which directly convert pitch to frequency and loudness to intensity of vibrations [8]. However, frequency response of these actuators overpass skin perception thresholds, so information embedded in high-frequency bands (i.e., over 1000 Hz) might be lost. Moreover, pitch discrimination is not constant and depends on frequency. The just noticeable difference (JND) between pitches varies as frequency varies; if frequency increases, the JND between pitches also increases. Thus, during design stages it would be important to consider that lower frequency bands will require a reduced pitch band, and vice versa [2]. If some frequencies are not perceivable because of a lack of intensity, the user may be provided with intensity control in an interface, such as in the Emoti-Chair [54], but independent control of intensity at a given frequency may require complex control protocols so that frequency remains between perceivable ranges. In addition, to overcome frequency limitations of touch, alternatives such as specialization of pitch have been proposed [32]. In [28] the authors named the spatialization of frequencies FM (Frequency Model). In the FM, audio signals have to be filtered to obtain frequency bands that are sent to different groups of actuators (see Figure 5). Results suggest that this method allows better perception of elements and emotional content of music than raw signals sent directly to the actuators. Moreover, in [39] the authors suggest that performance of the FM for hearing impaired users would increase if visual feedback is added. Consonance between pitches is another important feature of music, although dissonance can also be used as a composing resource. It has been studied in [57], and results show that users may process vibrotactile consonance in similar way as the auditory channel, supporting the results obtained in [64]. It was also found that evaluation of consonance enhances when vibrotactile feedback is presented in a wider area, which agrees with what was stated in [34]. Another concept introduced in [22] to relate pitch with touch is the use of tactile metaphors. The authors found that association between tactile metaphors such as sharpness, roughness, softness, weight, heat, and wetness, and musical characteristics such as pitch, loudness, timbre, and its combinations, is in fact possible. For instance *“higher pitches may be described as sharper, rougher, harder, colder, drier and lighter than lower pitches”*. Tactile metaphors might be of special value for the hearing impaired as they might have no experience with music but have experience with, for instance, textures that may be rendered or even synthesized from scratch, as suggested in [16]. Although, tactile metaphors remain unexplored as a method to map musical information to vibrotactile stimuli, they represent opportunities for future research.

### 4.3. Tactile Rendering of Melody

**Figure 6:**
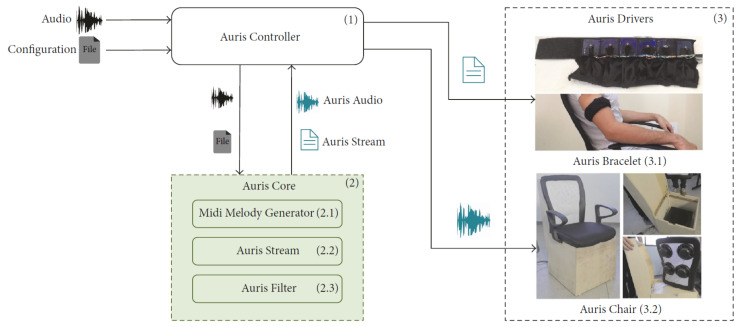
The Auris system for tactile rendering of melodic content, retrieved from in [40].

Melody builds up as a suitable combination of pitch changes over time. Therefore, most of the limitations for pitch conversion also apply to melody. Strategies already summarized for vibrotactile pitch may be combined to render vibrotactile melody. For instance, spatialization of frequencies in different parts of the body varying on time, will result in a practical representation of melody [20], and may include the implementation of spatio-temporal tactile illusions such as phantom motion [65]. However, the lack of frequency content will remain. Such as for rhythm, pitch and melody may be synthesized using software instruments from DAWs [47], but the lack of signal control will also remain, as well as frequency constrains due to perceptual limitations of touch. It might be more valuable to explore specific characteristics of melody to render it effectively to vibrotactile stimuli. For instance, one important characteristic of melody is interval, or the distance between notes. In [69], it was found that participants are able to discriminate intervals with changes in frequency of about 8 Hz, but this value depends on the location of the vibrotactile stimuli. The authors conclude that it is easier for participants to recognize larger intervals than smaller ones, which suggests that touch resolution for pitch discrimination is lower than that of the auditory. Another more complex strategy is to extract melodic content of audio and perform signal processing. One clear methodology implemented to extract melodic content of music and translate it to vibrotactile stimuli is presented in [40]. It consists on using algorithms to extract melodic features of audio (i.e., music information retrieval) and convert them to a MIDI (Musical Instrument Digital Interface) representation that then is executed, audio file is filtered and finally sent to the actuators contained in a bracelet named the Auris Bracelet. One key aspect of this HMP-H is the use of the Auris Bracelet to present melodic content along with the Auris Chair which presents vibrotactile feedback from filtered audio, as shown in Figure 6; this combination allows to convey musical information even encoded in frequencies over 1 kHz, as suggested by the researchers. Translating pitch and melody represents a challenge due to the tonal content of these musical features, and even more for hearing impaired users who may have a completely different conception of what music is [8].

### 4.4. Tactile Rendering of Timbre

**Figure 7:**
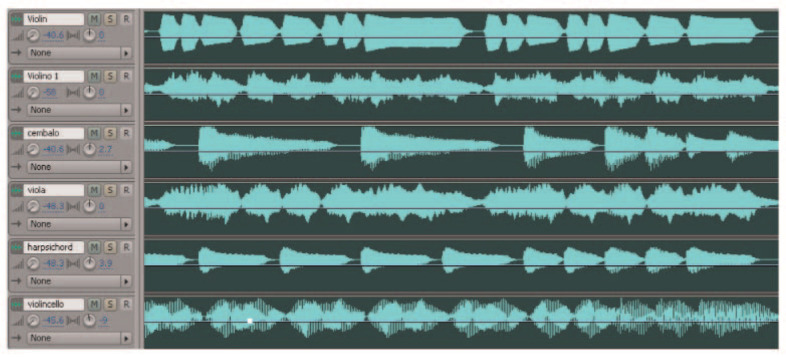
Different instrument tracks for TM implementation in the Model Human Cochlea, retrieved from in [14].

Timbre allows the listener to differentiate between tones played from one or another musical instrument. Timbre relies on the frequency content (i.e., spectral content) of audio signals, and therefore tactile rendering represents a challenge. Although the reduced tactile perception band will affect the recognition of small spectral content variations, individuals are able to recognize timbre of rendered audio signals from different musical instruments (e.g., piano, cello, or trombone) with vibrotactile stimuli only [79]. Moreover, the sense of touch is able to recognize waveform of signals [15], where the mechanoreceptors work as tactile filters that aid in the process [6]. This recognition ability may be used as a tool to render texture of sound (i.e., timbre) as vibrotactile texture. In [79], the researchers rendered different signals varying waveform, envelope, fundamental frequency, harmonics, duration, and ISOA, and found that individuals are able to differentiate these representations of timbre; participants with hearing impairment present the same ability. Another innovative method to render timbre was proposed in [32], where the researchers measure the amount of noise present in the audio signal, what they call a bark-based spectral flatness measure, and use it to perform an interpolation between a 500 Hz sine tone and a white noise signal. The result is a vibrotactile signal that represents timbre. However, evaluation of participants did not focus on timbre discrimination but on overall quality of the vibrotactile stimuli, so further exploration would be required. As earlier stated, timbre is closely associated with type of musical instrument. Such as for frequency, the authors of [28] proposed the TM (Track Model), a model that consists on splitting music into independent audio tracks, each containing different musical instruments, and then send the signals to groups of actuators (see Figure 7). Although this model ignores the frequency content that defines timbre of instruments, the researchers demonstrated that evaluation of perception was better than that obtained with the FM. The TM offers opportunities for future work as demonstrated by Hashizume et al. [38] who tested the method in a multi-modal experimental setup. Tactile metaphors is another resource that represent an opportunity for timbre rendering but, as mentioned in Section 4.2, this method remains unexplored. Thus, it is clear that the parent audio signal as well as control over signal waveform aids to reach tactile rendering. Moreover, variations on signal envelope: attack, decay, sustain, and release (ADSR), may offer a wider set of opportunities to obtain high fidelity vibrotactile rendering of timbre. Innovative methods may be proposed but it is necessary to present evidence that supports successful tactile rendering.

### 4.5. Tactile Rendering of Loudness

In traditional music notation loudness is a key feature to consider [65]. Subjectively, vibrotactile loudness is a variable corresponding to the distance that the skin is displaced by the stimuli [2]. Tactile rendering of loudness may be straightforward as can be mapped directly to the intensity of the actuators [8], and the use of software instruments and MIDI representations allows easy control [55]. Representation of loudness may even be enhanced by adding visual feedback in the form of brightness variation of light [18]. On the other hand, there are psychophysical implications that may affect the interaction between loudness and music tactile rendering. According to the work in [2], loudness is independent of frequency in the range of 20 to 40 Hz, while in [20] it was found that perception of loudness may be affected at low frequencies, which adds complexity to the definition of a suitable bandwidth to render musical information. In addition, according to Verillo [4], there are effects such as summation (i.e., perceptual increase of loudness) that occurs when two stimuli are presented in the same psychophysical channel (i.e., Pacinian or Non-Pacinian), or suppression (i.e., perceptual decrease of loudness) in a second vibrotactile stimuli when two tones are presented in independent psychophysical channels, which represents additional complexity for tactile rendering of loudness, specially when the HMP has arrays of actuators that present multiple vibrotactile stimuli.

## 5. Vibrotactile Music Composition (VMC)

Although music for the ears follows not only well established but also evolving methodologies, VMC is still being explored. VMCs are usually dedicated to hearing impaired users and transmitted by means of HMPs, but may also be enjoyed by hearing individuals. In [15], the authors propose a list of features that may constitute the foundations of tactile compositions: frequency, duration, intensity, waveform, spectral content, and space. Most of these elements have already been studied in Section 4, thus this section focuses on describing the strategies that have been implemented to compile these features and create VMCs.

### 5.1. Tactile Illusions

A resource used to compose for the sense of touch are spatio-temporal tactile illusions. Although there is no concise relation of tactile illusions with any musical element, the apparent sensations that may be created in the skin using vibrotactile stimuli offers a conceptual tool to convey meaningful information to the user. Apparent movement or phantom motion, for instance, may be correlated to music dynamics, emotion, or musician movements such as movement in dance [15]. Tactile illusions have been explored in the context of VMC by various authors [3,15,21,27,33,65,67], who agree that tactile illusions have good potential as a compositional resource.

### 5.2. Real-Time VMC

**Figure 8:**
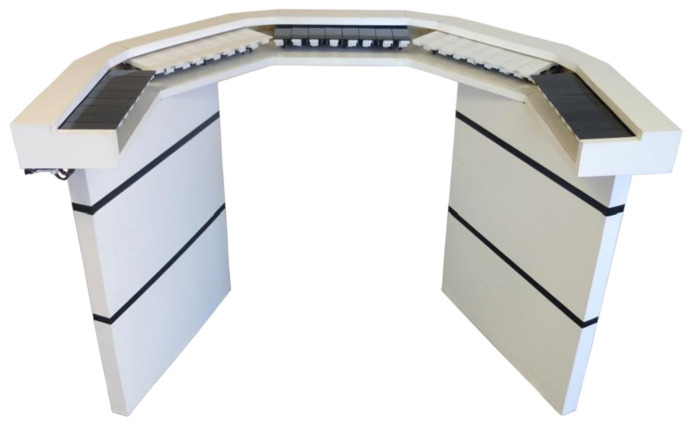
The Vibrochord, a vibrotactile musical input device VMID, retrieved from [66].

Real-time VMC has also been studied. In this case, a vibrotactile musical input device (VMID) is required. The VMID designed in [62], for instance, is a Nintendo Switch device with a tactile keyboard that renders vibrotactile signals presented to the performer by means of a VCA. One octave of notes are assigned to the keys from C-131 Hz to C-262 Hz that allows the performer to create complex vibrotactile compositions. Although there was no evidence to validate the performance of this VMID, it represents a suitable proposal for future exploration. Another device that has been designed is the Vibrochord [66] a piano-like VMID conceived exclusively to play vibrotactile compositions (see Figure 8). In this work, the authors propose an octave vibrotactile scale based on traditional western music, divided in numbers: 1—40 Hz, 2—56 Hz, 3—78.4 Hz, 4—109.76 Hz, 5—153.66 Hz, 6—215.12 Hz, 7—301.18 Hz, 8—421.65 Hz. To represent vibrotactile loudness, the keys of the Vibrochord respond to pressure, the harder they are played the higher is the intensity of vibrations, allowing the user greater expressiveness. The researchers have shown that vibrotactile music composition may require specific technology to allow more expressiveness and precision in real time playing. However, effects such as adaptation and learnability must be considered as the evaluation of performer or audience perception might be affected.

### 5.3. VMC from Scratch

**Figure 9:**
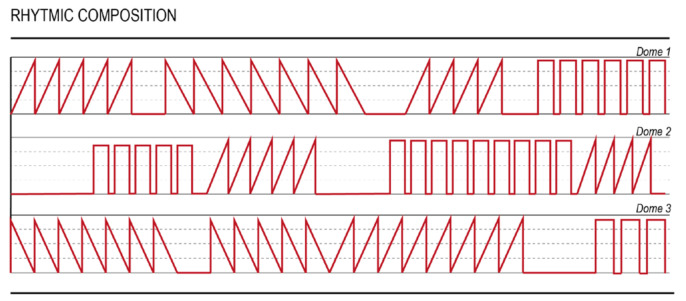
Example of rhythmic VMC created for the Hedonic Haptic Player, retrieved from in [35].

As mentioned before, some musical features may be rendered from scratch. This method was used by Vallgarda et al. in [35] to create VMCs that are played through the Hedonic Haptic Player [68]. The compositions were created in the basis of two musical features: rhythm and dynamics, rendered by modifying waveform, event length, wave amplitude, length in % of the original, silent event, and modulation frequency (see Figure 9). The researchers show how these compositions based on the interaction of vibrotactile stimuli, generates meaningful positive responses from users, more than just feeling one discrete vibration. Although the authors do not mention it, the results obtained suggest the perception of a combination of tactile illusions, which have already been considered as a powerful resource for VMC. Results obtained by Vallgarda et. al. are supported in [67], where VMCs were rendered to be played on a vibrotactile garment named Ilinx [80] and created from the point of view of artists. In [78], composers were provided with compositional resources and used them to build up tactile compositions. Resources comprise segments of signals with different waveforms (e.g., sine, saw, and square) and frequencies so that the artist creatively combines them to end up with an original tactile composition, that is later experienced by an audience in the Emoti-chair [54]. Although vibrotactile compositions were tested for audio visual environments, the method can be expanded to create VMCs for the hearing impaired; similar to what was implemented in [35,67].

## 6. Discussion and Conclusions

In this review, tactile rendering technology and methods to convey musical features through the skin have been systematically explored. The strategy to search relevant literature has been presented and the method used to manage documentation metadata was described. During the first stage of the review it was found that linking web-based tools such as Scopus, Mendeley, and Overleaf allows the researcher reliable and efficient organization of information.

Transmission of musical information through the skin has been accomplished by implementing vibrating technologies in devices whose design has evolved during the last twenty-eight years. The fundamental architecture of a HMP was described, and it was suggested that selection of actuators is important due to the availability of different types of them which have advantages and limitations regarding vibrotactile communication of musical elements. It was proposed that the ideal actuator should combine low-cost and lightweight design, good performance at low frequencies, and a balance between size and vibrotactile stimulation. VCAs and ERMs are the most used actuators (see Table 2, Table 3 and Table 4) and new concepts such as the DMA [37] and the string-motor device [42] are being studied. However, there are other technologies like mid-air ultrasound haptic feedback that demonstrated to be effective for frequency discrimination tasks [81] and may be investigated for touchless applications to explore music in museums or music stores and avoid disease transmission, for instance.

HMPs were classified depending on the way vibrotactile stimuli are presented to the user. Literature was organized into three groups: HMP installations (HMP-Is), wearable HMPs (HMP-WDs), and hybrid HMPs (HMP-H). Moreover, information was systematically allocated in tables according to year of publication, type of contact mechanism, type of actuator, type of stimuli presented, and musical features explored. Although preliminary research on HMPs was focused on fixed installations, it was found that ~54% of total publications by 2020 corresponds to HMP-WDs (see Figure 1), suggesting that HMP technology evolves towards wearability and portability. It is congruent not only with the evolution of electronics towards smaller and more efficient devices but also with the idea that listening to music is a day by day experience; something that everyone wants to enjoy anywhere at any moment. On the other hand, most of the installations such as chairs and platforms (see Table 2), bigger than a common portable music player, have been necessary to explore perception of various parts of the body simultaneously, and effects such as bone conduction and stimuli spatialization. Although these prototypes are neither wearable nor portable, they may evolve towards further application in medicine for instance to complement music therapy for the Alzheimer [82], dementia [83] or cancer [84,85] treatment; or entertainment such as amusement parks, movie theaters, video games, and virtual and augmented reality installations.

Regarding ATR, rhythm, loudness, and pitch are the predominant features explored in the literature as rendering seems to be straightforward (see Table 2, Table 3 and Table 4). Although direct rendering of pitch and loudness to frequency and intensity of vibrations is feasible, respectively, information may be lost due to frequency perception limitations of touch. Further, other musical elements such as melody and timbre require intensive signal processing such as music information retrieval techniques and in some cases the introduction of concepts such as spatialization of pitch or tactile metaphors, to avoid frequency discrimination limitations. Resources such as software instruments and MIDI representations may aid in the process. Thus, tactile rendering of musical features represents a challenge and a vast field for future research. Indeed, it seems to be clear that research on HMPs, and specifically ATR, has to consider that playing music is not only a whole complex performance perceived by the ear and the entire body through vibrations, but also the result of the summation of individual events, performances, instruments, colors, notes, rhythms and every musical feature. Therefore, music can be composed and decomposed, its vibratory nature and its relation with the human skin unveiled during this process, and might be different for the case of hearing impaired individuals who may perceive vibrotactile music in a different way.

Furthermore, a tactile composition was defined as a way to compose for the sense of touch. Strategies implemented to create VMCs were summarized and contrasted (see Table 2, Table 3 and Table 4). It was found that creating a compilation of sequences or patterns that change in time results more meaningful for users, instead of discrete stimuli presented solely. Some researchers used spatiotemporal tactile illusions with special focus on creating apparent movement that allowed composers and artists to expand creativity (see Section 5.1). VMCs may be created and later played through an HMP such as the Hedonic Haptic Player [35], the Emoti-chair [54] or the Ilinx [80], or may be played in real time using VMIDs such as the Vibrochord [66] or a mobile device (e.g., Nintendo Switch) [62]. VMC represents a new tool for composers. Nevertheless, more scientific evidence is required to overcome tactile perception limitations and psychophysical effects on users. VMC has been used as a novel resource for artists to compose for the sense of touch, and therefore an opportunity for hearing impaired users to perceive musical compositions by means of vibrotactile stimuli, but the lack of understanding of the human response to vibrotactile stimuli arrangements and the unavailability of suitable technology still restricts the development of novel applications. Accordingly, exploration of human skin limitations in the musical context should progressively be expanded to the use of more complex VMC arrangements and technologies.

Besides, vibrotactile music interaction between an audience and the performer has been explored, enabling a new communication channel to interact in a live concert installation [21]. This idea may be expanded towards interaction not only between the audience and the performer but also between groups of audiences, between individuals or even between concert venues, which opens up a new paradigm for event designers, musicians and artists in general.

During literature exploration, investigations with sound scientific foundation have been encountered with a clear hypothesis and clear objectives, which usually ends up unveiling the limitations of the human tactile sense or the pros and cons of some technology application. However, there are projects that seem to bypass this requirement and proceed to a conceptual and more aesthetic implementation, dismissing the limitations of the human perception capabilities and technology implications. It would be relevant to soon provide a clear general methodology for HMP research that enables effective and robust research to later develop a well-established starting point for technology development and implementation.

## Figures and Tables

**Table 1:** Vibrotactile channel characteristics, adapted from [2].

Psychophysical Channel	P	NP-I	NP-II	NP-III
Full name	Pacinian	Non-Pacinian I	Non-Pacinian II	Non-Pacinian III
Psychological type	FAF-II	FAF-I	SA-II	SA-I
Fiber innervation density (fingertip, per cm^2^)	21	140	49	70
Subjective sensation	“vibration”	“flutter”	*unknown*	“pressure”
Frequency range	40–500 Hz	2–40 Hz	100–500 Hz	0.4–3 Hz
Prime sensitivity range ${}^{1}$	250–300 Hz	25–40 Hz	150–400 Hz	0.4–1 Hz
Shape of frequency response function	U-shape	Flat ${}^{2}$	U-shape	Flat

${}^{1}$ Defined as best frequencies to lower threshold of perception; ${}^{2}$ Notch at 30 Hz.

**Table 2:** Overview of research on haptic music player installations (HMP-Is).

HMP-I	Year	Contact Mechanism	Type of Actuator	Stimuli	Features Explored
SOMATRON [52]	1992	Mattress	Speaker, Subwoofer	Vibrotactile, Auditory	Pitch
Vibratory Music (Patent) [45]	2002	Chair	N/A ${}^{1}$	N/A	N/A
Audiotactile Simultaneity [26]	2004	Chair	N/A	Vibrotactile, Auditory	ATFS ${}^{2}$
Symbolic Haptic Rendering [50]	2005	Knob	N/A	Vibrotactile	Tempo, energy
Model Human Cochlea (Design) [14]	2009	Chair	Voice coil	Vibrotactile, Auditory	FM ${}^{3}$, TM ${}^{4}$, VMLE ${}^{5}$
Multimodal reproduction [30]	2009	Seat	Voice coil	Vibrotactile, Auditory	VMLE, BRTF
Music Display and Haptic Chair [31]	2009	Chair	Speaker	Vibrotactile, Auditory	VMLE
Model Human Cochlea [53]	2009	Chair	Voice coil	Vibrotactile, Auditory	FM, TM, VMLE
Emoti Chair [54]	2010	Chair	Voice coil	Vibrotactile	FM, TM, VMLE
Whole Body Vibration [48]	2010	Chair	Voice coil	Vibrotactile, Auditory	ATFM ${}^{6}$, BRTF
Auditory-Tactile Music [29]	2013	Chair	Voice coil	Vibrotactile, Auditory, Visual	BRTF, VMLE
Haptic Display [55]	2013	Chair	Speaker	Vibrotactile, Auditory, Visual	VMLE,
Tactile Musical Device [32]	2015	Chair	Voice coil, Subwoofer	Vibrotactile	Loudness, Pitch, Rhythm, Timbre, VMLE
Skin Music [33]	2015	Chair	Voice coil	Vibrotactile, Auditory	VMLE
Musical Notes to the Skin [51]	2016	Button, platform	Voice Coil	Vibrotactile, Auditory, Visual	Pitch
Feeling the Beat [47]	2017	Platform	Voice coil	Vibrotactile, Auditory	Rhythm, Tempo, Beat Synchronization
Auditory-Tactile Experience of Music [25]	2018	Chair	Voice coil	Vibrotactile, Auditory	ATFS, BRTF, VMLE
Music with Vibrations [56]	2019	Chair	Voice coil	Vibrotactile, Auditory	F-EQ ${}^{7}$, VMLE,
Vibrotactile Consonance [57]	2019	Chair	Voice coil	Vibrotactile	MC ${}^{8}$
Haptic Music [49]	2020	Platform	Voice coil	Vibrotactile, Auditory, Visual	BRTF, Frequency, VMC ${}^{9}$, VMLE

${}^{1}$ Not applicable. ${}^{2}$ Audio-tactile frequency synchronism. ${}^{3}$ Frequency model: spatialization of frequencies. ${}^{4}$ Track model: spatialization of tracks. ${}^{5}$ Vibrotactile music with/without listening experience: explores overall perception of vibrotactile music, with or without music listening. ${}^{6}$ Audio-tactile frequency matching. ${}^{7}$ Frequency equalization: signal processing to control intensity of particular frequencies. ${}^{8}$ Melodic consonance. ${}^{9}$ Vibrotactile music composition.

**Table 3:** Overview of research on haptic music player-wearable devices (HMP-WDs).

HMP-WD	Year	Contact Mechanism	Type of Actuator	Stimuli	Features Explored
Cutaneous Grooves [15]	2003	Whole body suit	Voice coil, Subwoofer	Vibrotactile, Auditory	VMC
Model Human Cochlea [28]	2008	Belt	Speaker	Vibrotactile, Auditory	FM, TM, VMLE
Vibrotactile display [41]	2010	Belt	Speaker	Vibrotactile	FD ${}^{1}$
Vibrotactile Music System (Design) [66]	2011	N/A	N/A	N/A	VMLE
Vibrotactile Music System [66]	2012	Not available	Not available	Not available	FD, Interval Size, Pitch Direction
Dual Band HMP [37]	2013	Mobile device mock-up	DMA	Vibrotactile, Auditory	F-EQ, Rhythm
MUVIB [59]	2014	Bracelet	Voice coil	Vibrotactile, Auditory	Intensity
Vibrotactile Chords [64]	2014	Mobile device mockup	Voice coil	Vibrotactile	MC
Vibrotactile Composition [67]	2015	Jacket, leggins	ERM	Vibrotactile	VMC
CollarBeat [46]	2015	Collar, belt	Voice coil	Vibrotactile, Auditory	VMLE
Audio-Tactile Conversion [63]	2015	Mobile device mock-up	Voice coil	Vibrotactile, Auditory	FD
Vibroacoustic Device for Music [34]	2016	Belt	Motor-String, voice coil	Vibrotactile, Auditory	Amplitude, Frequency, Rhythm, VMLE,
MuSS-Bits [18]	2016	Bracelet, magnetic, belt	ERM	Vibrotactile, Visual	Rhythm, VMLE
Mood Glove [61]	2016	Glove	Voice Coil	Vibrotactile, Auditory, Visual	Rhythm, VMLE
Feeling Music [60]	2017	Bracelet	Voice coil	Vibrotactile, Visual	Rhythm
Hedonic Haptic Player: Design [68]	2017	Belt	Voice coil	Vibrotactile	VMC
Hedonic Haptic Player [35]	2017	Belt	Voice coil	Vibrotactile	Rhythm, VMC, VMLE
Hapbeat Test [42]	2017	Belt	Motor-String, voice coil	Vibrotactile, Auditory	Amplitude, Frequency, VMLE
Haptic Melodic Interval [69]	2018	Belt, mobile device mockup	Vibrotactile	Melodic interval	
Hapbeat Re-Design [70]	2018	Belt	Motor-String	Vibrotactile	N/A
Music Sensory Substitution System [19]	2018	Bracelet	ERM	Vibrotactile, Visual	Rhythm
Musical Scale Through Haptic Actuator [62]	2018	Mobile device mock-up, VMID	Voice coil	Vibrotactile, Visual	Melody, Pitch, Timming, VME
Musical Haptic Wearables [58]	2018	Belt (armband)	N/A	Vibrotactile, Auditory, Visual	API ${}^{2}$, VMLE
LIVEJACKET [38]	2018	Jacket	Piezoelectric, Subwoofer	Vibrotactile, Auditory	TM, VMLE
Musical Haptic Sleeve [39]	2019	Sleeve	Speaker	Vibrotactile, Auditory	VMLE
Body:Suit:Score [65]	2019	Whole body suit	ERM	Vibrotactile, Auditory	Pitch, FM, Tempo, VME
Tactile Identification in Music [71]	2019	Glove	Voice coil	Vibrotactile, Auditory	VMLE
Tactile Musical Emotion [72]	2020	Glove	Voice coil	Vibrotactile	Timbre, VMLE
Touching the audience [21]	2020	Bracelet, jacket	ERM	Vibrotactile, Auditory, Visual	API, VMLE
Vibrotactile Captioning [73]	2020	Glove	Voice coil	Vibrotactile, Auditory, Visual	EEG ${}^{3}$, Tempo, VMLE,
SENSE [74]	2020	Glove	Voice coil	Vibrotactile, Visual	VMLE

${}^{1}$ Frequency Discrimination; ${}^{2}$ Audience-Performer Interaction; ${}^{3}$ Vibrotactile perception supported with electroencephalogram.

**Table 4:** Overview of research on haptic music players-hybrid (HMP-H).

HMP-H)	Year	Contact Mechanism	Type of Actuator	Stimuli	Features Explored
Skinscape [27]	2001	Chair, bracelet	Voice coil, Subwoofer	Vibrotactile, Auditory	VMC
Vibrochord Vs. Piano [66]	2014	VMID + Chair	N/A	Vibrotactile	VMC, VME, VMLE
Vibrochord Design [76]	2014	Vibrotactile music input device + Chair	N/A	Vibrotactile	VMC, VME, VMLE
Concert for the Deaf [75]	2016	Platform, Jacket	Speaker	Vibrotactile, Visual	VMLE
Auris System [40]	2017	Chair, bracelet	Voice coil, Speaker	Vibrotactile	Frequency mapping, Rhythm, VMLE, EEG
Scaffolding the Music [8]	2018	Chair, belt	Chair with voice coil, Belt with ERM	Vibrotactile, Visual	Pitch, Frequency, Rhythm, VMLE

## Abbreviations

The following abbreviations are used in this manuscript (in order of appearance):

P	Pacinian
NP-I	Non-Pacinian I
NP-II	Non-Pacinian II
NP-III	Non-Pacinian III
FAF-I	Fast Afferent I
FAF-II	Fast Afferent II
ISOA	Inter-Stimulus Onset Asynchrony
ATR	Audio-Tactile Rendering
HCHI	Human–Computer Haptic Interface
SSS	Sensory Substitution System
DMI	Digital Musical Instrument
HAID	Haptic and Audio Interaction Design
HCI	Human Computer Interaction
HMP	Haptic Music Player
DAC	Digital to Analog Converter
VCA	Voice Coil Actuator
LRA	Linear Resonant Actuator
ERM	Eccentric Rotating Mass
DMA	Dual Mode Actuator
VMC	Vibrotactile Music Composition
HMP-I	Haptic Music Player - Installation
BRTF	Body Related Transfer Function
HRTF	Head Related Transfer Function
ATFS	Audio-Tactile Frequency Synchronism
FM	Frequency Model
TM	Track Model
VMLE	Vibrotactile Music with/without Listening Experience
ATFM	Audio-Tactile Frequency Matching
F-EQ	Frequency Equalization
MC	Melodic Consonace
HMP-WD	Haptic Music Player - Wearable Device
MHWA	Musical Haptic Wearables for Audiences
VR	Virtual Reality
AR	Augmented Reality
PWM	Pulse-Width Modulation
FD	Frequency Discrimination
API	Audience-Performer Interaction
EEG	Electroencephalogram
VMID	Vibrotactile Music Input Devices
JND	Just Noticeable Difference
MIDI	Musical Instrument Digital Interface
